# Adjuvant Effect of Cationic Liposomes for Subunit Influenza Vaccine: Influence of Antigen Loading Method, Cholesterol and Immune Modulators

**DOI:** 10.3390/pharmaceutics5030392

**Published:** 2013-07-25

**Authors:** Christophe Barnier-Quer, Abdelrahman Elsharkawy, Stefan Romeijn, Alexander Kros, Wim Jiskoot

**Affiliations:** 1 Division of Drug Delivery Technology, Leiden Academic Centre for Drug Research, Leiden University, P.O. Box 9502, RA Leiden 2300, The Netherlands; E-Mails: abdelrahman.sharkawy@hotmail.com (A.E.); romeijn@lacdr.leidenuniv.nl (S.R.); 2 Department of Soft Matter Chemistry, Leiden Institute of Chemistry, Leiden University, P.O. Box 9502, RA Leiden 2300, The Netherlands; E-Mail: a.kros@chem.leidenuniv.nl

**Keywords:** adjuvant, cationic liposomes, cholesterol, CpG, H3N2, hemagglutinin, imiquimod, immunogenicity, influenza

## Abstract

Cationic liposomes are potential adjuvants for influenza vaccines. In a previous study we reported that among a panel of cationic liposomes loaded with influenza hemagglutinin (HA), DC-Chol:DPPC (1:1 molar ratio) liposomes induced the strongest immune response. However, it is not clear whether the cholesterol (Chol) backbone or the tertiary amine head group of DC-Chol was responsible for this. Therefore, in the present work we studied the influence of Chol in the lipid bilayer of cationic liposomes. Moreover, we investigated the effect of the HA loading method (adsorption *versus* encapsulation) and the encapsulation of immune modulators in DC-Chol liposomes on the immunogenicity of HA. Liposomes consisting of a neutral lipid (DPPC or Chol) and a cationic compound (DC-Chol, DDA, or eDPPC) were produced by film hydration-extrusion with/without an encapsulated immune modulator (CpG or imiquimod). The liposomes generally showed comparable size distribution, zeta potential and HA loading. *In vitro* studies with monocyte-derived human dendritic cells and immunization studies in C57Bl/6 mice showed that: (1) liposome-adsorbed HA is more immunogenic than encapsulated HA; (2) the incorporation of Chol in the bilayer of cationic liposomes enhances their adjuvant effect; and (3) CpG loaded liposomes are more efficient at enhancing HA-specific humoral responses than plain liposomes or Alhydrogel.

## 1. Introduction

The main strategy against seasonal influenza outbreaks is vaccination. Subunit vaccines are known to be the safest influenza vaccines produced, but they are less immunogenic than whole virus and split vaccines. Some populations are less protected after vaccination, such as the elderly due to senescence of their immune system [1]. One way to enhance the immunogenicity of subunit vaccines is the use of adjuvants. 

Cationic liposomes are known for their ability to enhance the potency of subunit vaccines and may serve to lower the dose and thereby, enable the increase of vaccine supply. There is a large number of publications investigating cationic liposomes as an adjuvant for diverse antigens (reviewed in [2,3,4,5]). It has been demonstrated that the surface charge of the liposomes influences the immune response: positively charged lipid vesicles are taken up more efficiently than negatively charged or neutral vesicles by macrophages and dendritic cells (DCs) [6,7]. However, the different formulations, immunization schedules and read-out models used in these studies hamper straight comparisons and make it very difficult to judge which cationic liposomes have the most favorable adjuvant effect.

The work presented here is a follow up of our previous study in which we investigated the adjuvant effect of cationic liposomes mixed with a subunit H3N2 influenza vaccine based on purified hemagglutinin (HA) [8]. In that study, we showed that the adjuvant effect of cationic liposomes not only depends on their charge, but also on the cationic compound selected and its amount in the liposomal bilayer. The best adjuvant effect was obtained with liposomes containing DC-Chol (compared to DDA, eDPPC and DPTAP). However, it is not clear whether the tertiary amine head group and/or the sterol backbone of the DC-Chol molecule was responsible for this. 

The major aim of the present study was to get a better understanding of the superior adjuvant effect of DC-Chol liposomes. Ideally, we should compare liposomes made of DC-Chol with liposomes based on a cationic compound based on the same DC head group (a tertiary amine) but linked to a saturated carbon chain. Unfortunately, such a compound is not commercially available. Therefore, in the work presented here we focused on the influence of the Chol backbone in the cationic liposomal bilayer on HA immunogenicity by comparing the adjuvant effect of liposome formulations based on the same saturated cationic compounds mixed with either Chol or DPPC. Furthermore, we studied the effect of antigen loading method (encapsulation *versus* adsorption) and the encapsulation of immune modulators on the immune response against HA [9,10]. In general, co-delivery of an antigen with an immune modulator in one particulate system is an effective way to generate a strong immune response [11,12]. Moreover, the ability of cationic liposomes to enhance DC uptake could potentially help to target endosomal toll-like receptors (TLR), and for this reason we selected the following two TLR ligands: bacterial cytosine phosphodiester guanine oligomer (CpG), an agonist of TLR-9 [13], imiquimod, a TLR-7 agonist [14,15]. The immunogenicity of the liposomal HA formulations was evaluated for APC maturation *in vitro* as well as in a mouse model and compared to that of HA formulated with aluminum hydroxide (Al(OH)_3_), an adjuvant known to promote a Th2 type response.

## 2. Experimental Section

### 2.1. Materials

Cholesterol, 1,2-diacyl-*sn*-glycero-3-ethylphosphocholine (eDPPC), dimethyl dioctadecyl-ammonium bromide (DDA), 3β-[*N*-(*N*',*N*'-dimethylaminoethane)-carbamoyl] cholesterol (DC-Chol) were obtained from Avanti Lipids (Alabaster, AL, USA). 1,2-Dipalmitoyl-*sn*-glycero-3-phosphocholine (DPPC) was kindly provided by Lipoid GmbH (Ludwigshafen, Germany). Influenza hemagglutinin (HA) antigen (H3N2 Wisconsin strain) was obtained from Solvay (Weesp, The Netherlands). Bovine serum albumin (BSA) was purchased from Merck (Darmstadt, Germany). ELISA plates were obtained from Greiner (Alphen a/d Rijn, The Netherlands). Horseradish peroxidase (HRP) conjugated goat anti-mouse IgG (γ chain specific), IgG1 (γ1 chain specific) and IgG2a/c (γ2a chain specific) were ordered from Southern Biotech (Birmingham, AL, USA). Chromogen 3,3',5,5'-tetramethylbenzidine (TMB) substrate buffer for ELISA, granulocyte macrophage colony stimulating factor (GM-CSF) and interleukin-4 (IL4), were provided by Biosource-Invitrogen (Breda, The Netherlands). CpG (1826 and 2006) and imiquimod were purchased from InvivoGen (Toulouse, France). Alhydrogel was kindly provided by Brenntag (Frederikssund, Denmark). Fetal bovine serum (FBS) and all culture media, including penicillin/streptomycin and trypsin were supplied from Gibco (Invitrogen, Carlsbad, CA, USA). Nimatek^®^ (100 mg/mL ketamine, Eurovet Animal Health B.V., Bladel, The Netherlands) and Rompun^®^ (20 mg/mL xylazine, Bayer B.V., Mijdrecht, The Netherlands) were obtained from the pharmacy of Leiden University Medical Center.

### 2.2. Preparation of Cationic Liposomes

Liposomes were prepared by the film hydration method, followed by extrusion, as described previously [8]. Briefly, desired amounts of a cationic compound (DDA, eDPPC, or DC-Chol) were dissolved in a chloroform/methanol 9:1 (*v*/*v*) solution with a neutral phospholipid (DPPC) or with Chol, and mixed in a round bottom flask of 50 mL. A thin lipid film was formed at the bottom of this flask under reduced pressure by using a rotary evaporator. The film was hydrated in a HEPES sucrose buffer (20 mM HEPES, 10% (*w*/*v*) sucrose, pH 7.4) to obtain a final lipid concentration of 5 mg/mL. During the hydration step the temperature was maintained at 60 °C for 20 min, with continuous stirring at 300 rpm. The dispersion was extruded (LIPEX™ extruder, Northern Lipids Inc., Burnaby, Canada) 5 times through a polycarbonate filter (Nuclepore Track-Etched Membranes, Whatman, 's-Hertogenbosch, The Netherlands) with a pore size of 800 nm and 5 times through a filter with a pore size of 200 nm (Nucleopore Millipore, Amsterdam, The Netherlands). 

Loading of TLR ligands into the DC-Chol:DPPC liposomes was done in two different ways, depending on the TLR ligand:CpG was dissolved in the buffer used to hydrate the lipid film, while imiquimod was dissolved in a chloroform/methanol 9:1 (*v*/*v*) solution and mixed with the lipid solution before preparation of the lipid film. For both TLR ligands, a dose of 2 µg adjuvant/400 µg total lipids was used to obtain an adjuvant/antigen ratio of *ca*. 1:1 (*w*/*w*). 

### 2.3. Preparation of HA Formulations

HA was adsorbed to liposomes (HA/liposomes) as described before [8]. Briefly, the antigen stock solution (453 µg/mL HA) was mixed with the preformed liposomes to obtain a final concentration of 10 µg/mL HA (corresponding to 2 µg HA per injected dose) and 2000 µg/mL lipid compounds. Al(OH)_3_ formulations were prepared by diluting Alhydrogel with HEPES sucrose buffer. Subsequently, the antigen solution was added to an equal volume of adjuvant, to obtain a final concentration of 10 µg/mL HA and 600 µg/mL Al(OH)_3_. 

For the encapsulation of HA in DC-Chol:DPPC liposomes, we adapted a method described by Babai *et al*. [16]. Briefly the HA/DC-Chol:DPPC liposomes, produced with the adsorption method described above, were freeze-dried overnight, followed by a stepwise rehydration with warm (40 °C) Milli-Q water.

### 2.4. Characterization of the Formulations

#### 2.4.1. Hydrodynamic Diameter and Zeta Potential

Particles’ hydrodynamic diameter and polydispersity index (pdi) were determined by means of dynamic light scattering (DLS) using a NanoSizer ZS (Malvern Instruments, Worcestershire, UK). The zeta potential (ZP) of the liposomes was measured by laser Doppler velocimetry on the same instrument by using a zeta dip cell (Malvern Instruments, Worcestershire, UK). Prior to analysis, samples were diluted 10 fold in 20 mM HEPES, pH 7.4. The measurements were performed at 25 °C and Malvern DTS software (version 6.10, Worcestershire, UK) was used for data acquisition and analysis.

#### 2.4.2. HA Loading

HA was labeled with IRDye 800 CW (Licor Bioscience, The Netherlands) according to the manufacturer’s instructions and the labeled HA (IR-HA) was used to estimate the extent of antigen adsorption to the cationic liposomes. The IR-HA was mixed with the cationic liposomes (2 µg IR-HA/400 µg total lipids). HA adsorption to the cationic liposome was measured via cation-exchange chromatography on a Hi Trap CM FF column (GE Healthcare, Pittsburgh, PA, USA) and measuring the fluorescence intensity of the unbound fraction, as described before [8].

#### 2.4.3. Adjuvant Loading

The amount of CpG incorporated in the liposomes was indirectly determined by using FITC-labeled CpG (10% of total CpG). The free TLR ligand was separated from the liposomes by filtration using a Vivaspin 2 centrifugal concentrator (PES membrane, MWCO 300 kDa, Sartorius Stedim, Nieuwegein, The Netherlands) and quantified using a fluorescence plate reader (TECAN infinite M1000, Tecan Group Ltd., Männedorf, Switzerland). The same separation method was used for the imiquimod-containing liposomes and (unlabeled) imiquimod was quantified by its absorbance at 247 nm.

### 2.5. Immunogenicity Study

Female C57-BL/6 mice, 8 weeks old at the start of the vaccination study, were purchased from Charles River (Maastricht, The Netherlands) and maintained under standardized conditions in the animal facility of the Leiden Academic Centre for Drug Research at Leiden University. The study was done under the guidelines compiled by the Animal Ethic Committee of The Netherlands. The mice received two subcutaneous injections of 200 µL vaccine containing 2 µg HA: a prime (day 1) and a boost (day 22). Blood samples were taken one day before prime and boost, and 3 weeks after the boost. Hemagglutination inhibition (HI) titers in serum after boost were determined as described previously [8]. IgG isotype-specific analysis was performed by ELISA using the horseradish peroxidase-conjugated anti-mouse total IgG, IgG1 and IgG2a. C57BL/6 mice express the *Igh1-b* gene, which encodes the IgG2c isotype rather than IgG2a. However, here we used an anti-IgG2a isotype (which cross-reacts with IgG2c [17]) and titers are reported as IgG2a/c titers. Antibody titers were determined at the midpoint of the optical density-log dilution curves after subtraction of the naïve background, and none-responding mice were given an arbitrary titer of 10. Furthermore, mouse spleens were collected three weeks after the last immunization, and after homogenization the cells were re-stimulated *in vitro* with 5 µg/mL of HA, while the release of interferon gamma (IFN-γ) was determined by ELISA.

### 2.6. *In Vitro* Uptake of HA by Dendritic Cells

HA was conjugated with FITC by using the FluoReporter^®^ FITC Protein Labeling Kit (Invitrogen, Paisley, UK) according to the manufacturer’s instructions. Immature DCs were incubated for 4 h (at 4 °C and 37 °C) with to 2.5 µg/mL HA-FITC, free or adsorbed to 50 µL of a 2 mg/mL liposome suspension. The cells were washed three times with PBS containing 1% (*w*/*v*) bovine serum albumin and 2% (*v*/*v*) fetal bovine serum). Next, HA-FITC association with the DCs was quantified by flow cytometry (FACSCanto II, Becton Dickinson, San Jose, CA, USA). Living cells were gated based on forward and side scatter and HA-FITC association was expressed as the mean fluorescence intensity (MFI).

### 2.7. *In Vitro* Dendritic Cell Maturation

Monocytes isolated from buffy coats (purchased from Sanquin, Leiden, The Netherlands) were cultivated to differentiate into immature dendritic cells (DCs), as described previously [8]. Briefly, immature DCs were incubated for 48 h at 37 °C in 1 mL cell culture medium in presence of 10 µL of a 2 mg/mL liposome suspension. After being washed three times the cells were incubated for 30 min with a mixture of 50× diluted anti-MHCII-FITC or anti-CD86-APC, anti-CD40-PE (Becton Dickinson, Breda, The Netherlands) on ice. The expression of the surface markers was quantified by using flow cytometry (FACS canto, Becton Dickinson). Live cells were gated based on forward and side scatter. The up-regulation of the three surface markers by 100 ng/mL LPS (positive control) was set at 100%. At least 10,000 gated cells were analyzed in each experiment.

### 2.8. Statistical Analysis

Antibody and HI titers were logarithmically transformed before statistical analysis. All data were analyzed by a two-tailed Mann-Whitney test to demonstrate significant differences between the experimental groups, except for Figure 4, Figure 5 where the increase of the immune responses induced by DPPC:DC-Chol liposomes + immunomodulators was compared to the negative control (DPPC:DC-Chol liposomes alone), and thus we used a one-tailed Mann-Whitney test.

## 3. Results

### 3.1. DC-Chol:DPPC Liposomes with Adsorbed *versus* Encapsulated HA

We prepared DC-Chol:DPPC liposomes loaded with the antigen HA using two different methods: adsorption (HA ad./DC-Chol:DPPC) and encapsulation (HA enc./DC-Chol:DPPC). After preparation, the particle size, the zeta-potential and the HA loading of each formulation were determined. The results (Table 1) showed that the size and pdi of the HA/liposomes were similar (also compared to the empty DC-Chol:DPPC liposomes). Additionally, the HA loading efficiency measured was very similar for the two HA loading methods (60% *versus* 63%). Finally, the positive ZP of the liposomes tended to become smaller with the encapsulation or adsorption of HA.

**Table 1:** Physicochemical characteristics of DC-Chol:DPPC liposomes before and after hemagglutinin (HA) loading.

Formulation	Lipid molar ratio	HA/liposomes			
		Z_ave_ (nm)	pdi	ZP (mV)	LE HA (%)
DC-Chol:DPPC	1:1	160 (±4)	0.07 (±0.01)	+51.1 (±4.5)	not applicable
HA ad./DC-Chol:DPPC	1:1	165 (±5)	0.08 (±0.02)	+41.8 (±9.5)	60 (±4)
HA enc./DC-Chol:DPPC	1:1	155 (±18)	0.08 (±0.04)	+46.5 (±2.3)	63 (±5)

Results are expressed as average ± standard deviation (*n* = 3 independent batches).

**Figure 1:**
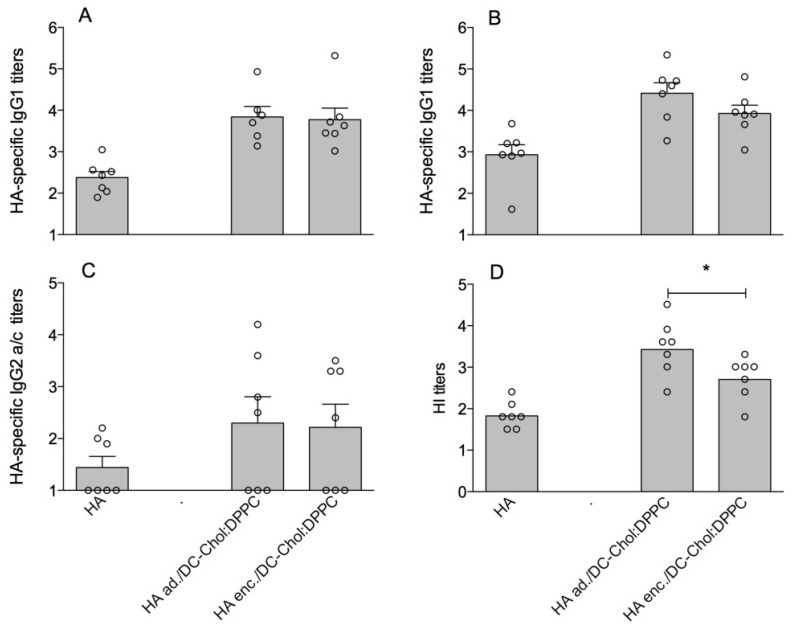
Immune response in mice vaccinated with 2.0 µg of free HA *versus* HA adsorbed to DC-Chol:DPPC liposomes (HA ad./DC-Chol:DPPC) and HA encapsulated in DC-Chol:DPPC liposomes (HA enc./DC-Chol:DPPC): HA-specific serum IgG1 titer after prime (**A**) and boost (**B**); IgG2a/c titer after boost (**C**); and HI titer after boost (**D**). For panels A–C, each dot represents the log serum titer of an individual mouse (non-responding mice were given an arbitrary titer of 10) and bars represent average log titer + SEM. For panel D, each dot represents the log HI titer in serum of an individual mouse and bars represent the geometric mean. Significant differences between the groups treated with liposomal formulations are indicated with * (*p* < 0.05).

To assess the immunogenicity of the two liposomal HA formulations (HA ad./DC-Chol:DPPC and HA enc./DC-Chol:DPPC), these were injected subcutaneously in mice. HA-specific serum IgG1, and IgG2a/c titers were assessed after the first (prime) and the second (boost) immunization, and hemagglutination inhibition (HI) titers, as a measure for the level of functional antibodies, were measured after the boost. The results (Figure 1) show a superior HI titer induced by the HA adsorbed liposomes after the boost immunization compared to the HA encapsulated liposomes (*p* < 0.05) (Figure 1D). The IgG1 titers measured after the boost showed the same trend (Figure 1B), although the difference was not statistically significant. No difference was observed between the IgG2a/c titers induced by the two formulations (Figure 1C). In conclusion, HA ad./DC-Chol:DPPC liposomes were slightly more immunogenic and are easier to prepare than HA enc./DC-Chol:DPPC liposomes. Therefore, we followed the adsorption procedure in the follow-up studies described below.

### 3.2. Cationic Liposomes with Different Bilayer Compositions

A series of liposome formulations was prepared to investigate the influence of the Chol backbone on the adjuvant effect of cationic liposomes. As detailed earlier, we were not able to use the DC-Chol for this study as there are no commercially available compounds with the same head group linked to saturated carbon chains. Instead, we prepared cationic liposomes either composed of (50 mol% Chol + 50 mol% cationic compound) or (50 mol% neutral saturated phospholipid + 50 mol% cationic compound). For this study, two cationic compounds were selected: DDA and eDPPC (the two best cationic compounds after DC-Chol identified in our previous study [8]); and the neutral saturated phospholipid DPPC. The four resulting formulations enabled the comparison of cationic liposomes containing either 100% saturated chain liposomes or 50% Chol.

Using the HA adsorption method, the antigen was mixed with each type of liposomes and the physicochemical characteristics of the resulting formulations were determined, as summarized in Table 2. In line with our previous paper [8], the addition of HA to the four liposome formulations induced an increase in the liposome’s hydrodynamic diameter (from 2 to 31 nm) and a slight decrease of their ZP (data not shown). These results indicate that the negatively charged antigen was successfully adsorbed to the surface of the cationic liposomes. The adsorption of HA to the liposomal surface was confirmed by measuring the retention of HA during cation exchange chromatography, showing HA loading between 68% and 79% (Table 2). Apart from the difference in pdi noticed for the DDA:DPPC formulation, likely due to some aggregation, the formulations were similar in size and ZP, and showed a similarly strong interaction with the antigen, irrespective of the presence of Chol. Furthermore, differential scanning calorimetry showed no sharp transition, corresponding to the gel-to-liquid-crystalline phase transition temperature of the saturated lipid, for liposomes containing DC-Chol or Chol. In contrast, sharp transitions were detected at 40 °C and 55 °C for liposomes prepared from DDA:DPPC and eDPPC:DPPC, respectively (data not shown). These observations are consistent with our previous results [8].

**Table 2:** Physicochemical characteristics of cholesterol- and DPPC-based cationic liposomes after mixing with HA.

Formulation	Molar ratio	HA/liposomes			
		Z_ave_ (nm)	pdi	ZP (mV)	LE HA (%)
DDA:DPPC	1:1	207 (±11)	0.44 (±0.02)	+44 (±3.3)	76 (±2)
DDA:Chol	1:1	179 (±2)	0.09 (±0.01)	+46.5 (±1.2)	78 (±3)
eDPPC:DPPC	1:1	150 (±5)	0.13 (±0.02)	+47.8 (±1.4)	68 (±3)
eDPPC:Chol	1:1	166 (±4)	0.10 (±0.02)	+45.5 (±0.9)	79 (±1)

Results are expressed as average ± standard deviation (*n* = 3 independent batches).

**Figure 2:**
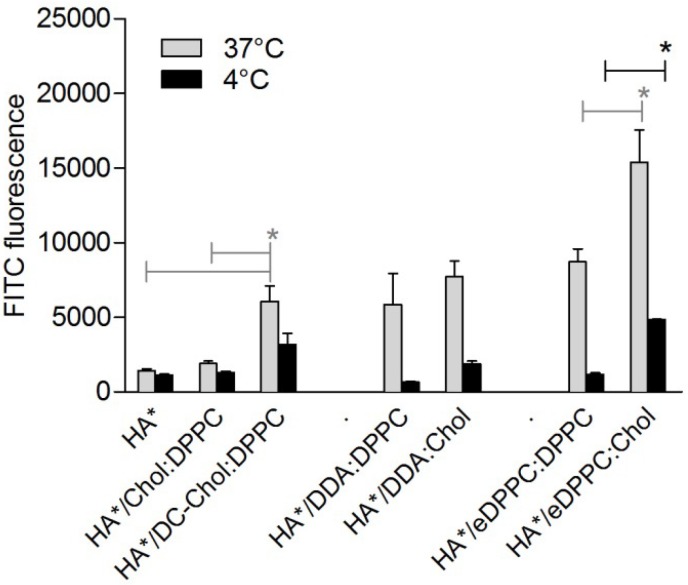
Effect of formulations on HA-FITC (indicated as HA*) uptake by DCs. Bars represent the mean fluorescence intensity (+SEM) of three batches of DCs incubated with HA-FITC alone or mixed with liposomes for 4 h at 37 °C *versus* 4 °C. Significant differences between the formulations are indicated with * (*p* < 0.05). See Supplementary Material, Figure S1, for representative side scatter-forward scatter dot plots.

We investigated if the cationic liposome composition influences the interaction between the antigen and monocyte derived DCs. For this purpose we produced a HA-FITC conjugate (referred to as HA*), which was adsorbed to different cationic liposomes formulations, and monitored the relative amount of DC associated HA* with flow cytometry. Figure 2 shows that after 4 h incubation at 37 °C, the DCs mixed with positively charged liposomes (HA*/DC-Chol:DPPC) showed a higher MFI compared to the groups incubated with either HA* alone (*p* < 0.05) or HA* mixed with a neutral liposome formulation (HA*/DPPC:Chol). Not only the liposome’s charge, however, affected HA’s interaction with the DCs, but also the presence of Chol in the cationic liposomes: incubation of DCs with HA*/eDPPC:Chol resulted in a higher MFI than DCs incubated with HA*/eDPPC:DPPC. Besides, for DDA liposomes, the MFI induced by HA*/DDA:Chol tended to exceed that induced by HA*/DDA:DPPC, but no statistical significance was demonstrated. 

With the above incubation study we assessed HA* association with DCs (*i.e.*, the sum of adhesion and uptake). In order to study the extent of HA* uptake by DCs, the incubation study was also performed at 4 °C, where energy dependent uptake is inhibited but adhesion will still occur [18]. DCs incubated with the cationic HA*-containing liposomes at 4 °C showed a lower MFI compared to the 37 °C groups, indicating that the cell-associated fluorescence at 37 °C was mainly caused by HA* uptake rather than association with the cell membrane. In contrast, DCs incubated with HA* alone or mixed with neutral liposomes showed hardly any decrease in MFI compared to the 37 °C conditions, indicating that most of the (low) MFI intensity at 37 °C was due to adhered HA*.

**Figure 3:**
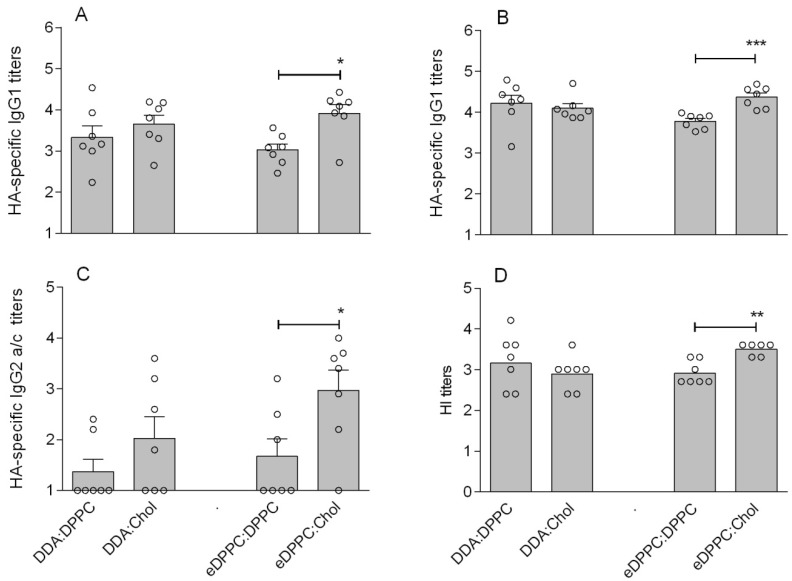
Immune response in mice vaccinated with 2.0 µg HA, either free or mixed with liposomes with or without Chol: HA-specific serum IgG1 after prime (**A**) and boost (**B**); IgG2a/c after boost (**C**); and HI titer after boost (**D**). For panels A–C, each dot represents the log serum titer of an individual mouse (non-responding mice were given an arbitrary titer of 10) and bars represent average log titer + SEM. For panel D, each dot represents the log HI titer in serum of an individual mouse and bars represent the geometric mean. Significant differences between the groups treated with the liposomal formulations are indicated with * (*p* < 0.05), ** (*p* < 0.01), or *** (*p* < 0.001).

The immunogenicity of the HA loaded cationic liposomes was assessed in mice. Figure 3 shows that the presence of Chol positively influenced the immune response against HA. In particular, eDPPC:Chol liposomes induced superior IgG1 titers after prime (*p* < 0.05) and boost (*p* < 0.001), as well as superior IgG2a/c and HI titers after boost (*p* < 0.05 and *p* < 0.01, respectively), compared to eDPPC:DPPC liposomes. The influence of Chol was not as clear for the DDA liposomes: DDA:Chol liposomes induced either a similar (IgG1 and HI titer) or a slightly but not significantly higher (IgG2a/c) immune response compared to liposomes without Chol (DDA:DPPC). These results are in line with the *in vitro* DC studies discussed above and suggest an overall positive effect of the presence of Chol in the liposomal bilayer on the adjuvanticity of the cationic eDPPC liposomes.

### 3.3. Encapsulation of Immune Modulators in DC-Chol:DPPC Liposomes

We prepared DC-Chol:DPPC liposomes with two different immune modulators: CpG and imiquimod (10 µg/mL). This resulted in three formulations with similar physicochemical characteristics and the addition of HA had little effect on their size, while a small drop of the ZP was observed (data not shown). The characteristics of the liposomes after mixing them with HA are summarized in Table 3. The average size of the adjuvanted HA/liposomes ranged between 165 and 177 nm and the average ZP between +43.4 and +47.2 mV. The incorporation of imiquimod in the DC-Chol:DPPC bilayer did not affect the HA loading efficiency. However, the encapsulation of CpG in the liposomes induced a drop of the HA loading efficiency from 60% (Table 1) to 30% (Table 3), probably due to competition between the antigen and the TLR ligand, both of which are negatively charged. The loading efficiency of CpG and imiquimod was practically 100%.

**Table 3:** Physicochemical characteristics of aluminium hydroxide and adjuvanted cationic liposomes after mixing with HA.

Formulation	Molar-ratio	HA/liposomes (HA/alum)				
		Z_ave_ (nm)	Pdi	ZP (mV)	LE adj (%)	LE HA(%)
Al(OH)_3_	-	5,624 (±543)	0.34 (±0.09)	+2.7 (±0.3 )	-	-
DC-Chol:DPPC + CpG	1:1	177 (±4)	0.10 (±0.04)	+46.2 (±0.6)	99	30 (±9)
DC-Chol:DPPC + Imiquimod	1:1	168 (±3)	0.09 (±0.02)	+43.6 (±2.6)	100	60 (±2)

Results are expressed as average ± standard deviation (*n* = 3 independent batches).

**Figure 4:**
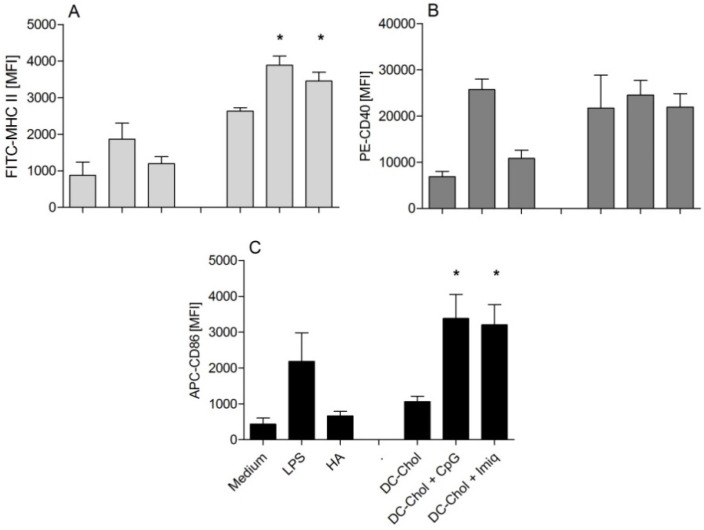
Upregulation of DC maturation markers induced by free HA *versus* HA mixed with liposomes: MHCII (**A**); CD40 (**B**); and CD86 (**C**). The values are expressed as percentage of mean fluorescence intensity (compared to a 100 ng/mL LPS control group, arbitrarily set as 100%). Error bars represent SEM (*n* = 3). Significant differences between the formulations and the DC-Chol:DPPC group are indicated with * (*p* < 0.05). See Supplementary Material, Figure S2, for representative side scatter-forward scatter dot plots.

In our previous study, DC-Chol:DPPC liposomes showed a relatively weak ability to activate DCs, in spite of their adjuvant effect demonstrated *in vivo* [8]. Since imiquimod and CpG have been described in the literature as potent immune modulators for DCs’ activation [19,20], we investigated the potency of these two immune modulators incorporated in HA/DC-Chol:DPPC liposomes to enhance DC maturation. After 48 h of incubation, the DCs incubated with HA/liposome formulations containing immune modulators showed a significantly higher level (*p* < 0.05) of MHCII expression compared to DCs exposed to HA/DC-Chol:DPPC liposomes (Figure 4A). A comparable effect (*p* < 0.05) was observed for CD86 (Figure 4C), but not for CD40 (Figure 4B). These results indicate that encapsulating immune modulators in DC-Chol:DPPC liposomes enhances their adjuvanticity, as shown by the increased MHCII and CD86 expression indicative of DC activation [21]. 

The liposomal formulations were tested for their immunogenicity in mice in comparison with HA/DC-Chol:DPPC liposomes and HA adjuvanted with the licensed adjuvant: aluminum hydroxide (HA/Al(OH)_3_) [22]. 

**Figure 5:**
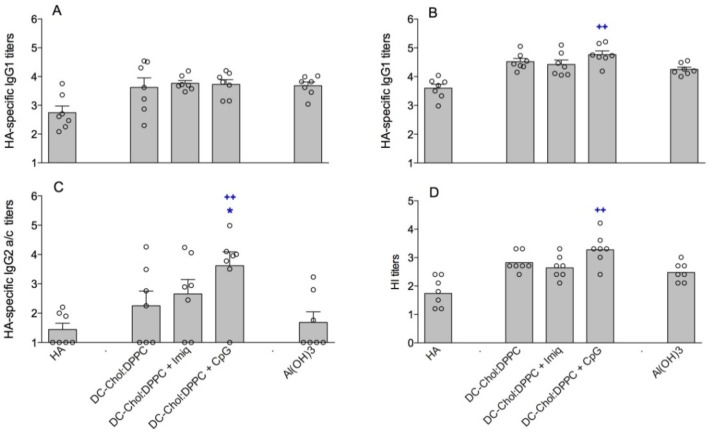
Immune response in mice vaccinated with 2.0 µg HA, free or mixed with Al(OH)_3_ or with liposomes containing different immune modulators: HA-specific serum IgG1 after prime (**A**); and boost (**B**); IgG2a/c after boost (**C**); and HI titer after boost (**D**). For panels A–C, each dot represents the log serum titer of an individual mouse (non-responding mice were given an arbitrary titer of 10) and bars represent average log titer + SEM. For panel D, each dot represents the log HI titer in serum of an individual mouse and bars represent the geometric mean. Significant differences between the liposomal formulations and the DC-Chol:DPPC group are indicated with * (*p* < 0.05); significant differences between the liposomal formulations and the Al(OH)_3_ group are indicated with **^++^** (*p* < 0.01).

Our results showed CpG increased the immunogenicity in mice of the HA/liposomes, whereas imiquimod appeared to be ineffective (Figure 5). The incorporation of CpG in the HA/DC-Chol:DPPC liposomes resulted in a raise of the IgG2a/c titer and the number of responders compared to the HA/DC-Chol:DPPC liposomes alone (*p* < 0.05). Also the HI titer seemed to be increased, although not significantly (*p* = 0.0548). When compared to HA alone, Al(OH)_3_ enhanced the anti-HA IgG1 titers (Figure 5A,B) and HI titers (Figure 5D), but not the IgG2a/c titers (Figure 5C). This is in line with aluminum salts known ability to enhance a Th2 type response [22]. The liposomal CpG formulation, however, not only induced significantly higher IgG1 and HI titers but also elicited higher IgG2a/c titers after the boost compared to HA/Al(OH)_3_ (Figure 5B–D). In contrast, CpG alone (without liposomes) when mixed with HA did not induce a stronger immune response compared to HA/DC-Chol:DPPC liposomes or HA/Al(OH)_3_ (unpublished results). This, together with the above-mentioned results, illustrates that encapsulation of CpG in cationic liposomes is advantageous for their adjuvant effect.

## 4. Discussion

In the present study, adsorption of HA to cationic DC-Chol:DPPC liposomes was shown to be a simple and effective method to enhance the immunogenicity of the antigen, as compared to liposomal encapsulation of the antigen. The higher HI titers induced by adsorbed HA compared to encapsulated HA might be explained by the presentation of the antigen at the outer surface of the liposomes, as previously detailed for a virosomal vaccine composed of influenza membrane fragments (including HA) mixed with phospholipids. It was shown that the positioning of the antigen in the virosomes influenced its processing and presentation pathway [23]. Although encapsulated and surface-associated liposomal antigens may induce T cell responses equivalently, several studies have shown increased antibody induction mediated by surface-associated antigen [24,25,26]. This may be because surface-associated antigen is available on the particle surface for antibody or B cell receptor recognition, whereas encapsulated antigen requires vesicle disruption to become accessible [27,28]. For surface-associated antigens, B cells may recognize intact liposomal antigen directly or via opsonized liposomes bound to Fc receptors or complement receptors on APCs [29]. Another hypothesis is that the way HA is associated with the liposomes could induce different processing of the antigen and a different kinetics of the anti-HA antibody response. 

In our previous study, DC-Chol:DPPC liposomes were shown to be a stronger adjuvant for HA than cationic DPPC liposomes containing other cationic compounds (DDA, DPTAP, or eDPPC) [8]. However, it remained unclear whether the cationic tertiary amine (DC-) head group or the Chol backbone was responsible for the superior adjuvant effect. The results of our present study indicate that the presence of Chol contributes to the adjuvant effect of cationic liposomes. In particular, the HA/eDPPC:Chol liposomes enhanced both the antigen uptake by DCs *in vitro* and the immunogenicity of HA (HI titer, IgG2a/c and IgG1 antibody secretion) compared to the liposomes without Chol (HA/eDDPC:DPPC). A positive correlation between uptake by APCs and adjuvanticity *in vivo* has been described previously [30]. However, for the DDA-liposomes we noticed only a small influence of Chol, although there was a trend that the presence of Chol in DDA-liposomes increased HA uptake *in vitro* and enhanced the IgG2a/c response *in vivo*. 

The influence of Chol in liposomal formulations for vaccination has been investigated in some other studies. For instance, in a study about the relationship between the phospholipid composition and the immunogenicity of a liposomal tumor antigen, Bakouche *et al.* showed that optimal immunogenicity in rats was obtained with 20 mol% Chol in the liposomal bilayer [31]. Batenjany *et al*. [32] reported that the immunogenicity of a Muc1 mucin peptide in DPPC/Chol liposomes for immunotherapy of adenocarcinoma was optimal when the Chol content was above 30 mol%. Other studies also showed a beneficial effect of Chol on vaccination [33], but it is still not clear which mechanism is responsible for this effect. Considering that cell membranes contain about 25 mol%–50 mol% of Chol, this lipid could play an important role in the interaction between cells and liposomes [28]. Furthermore, Chol is known to influence membrane fluidity and to enhance liposomal stability. For instance, the interaction of liposomes with plasma proteins has no negative effect on liposome stability when they are enriched in Chol [29], which is explained by its influence on the lipid packing in the liposomal membrane and its ability to prevent phospholipid loss due to uptake by high density lipoproteins [30]. 

Regarding the different antibody subtypes, we noticed a raise in the anti-HA IgG2a/c response after immunization with HA/eDPPC:Chol liposomes, which are known to modulate the immune response to a Th1 direction [34]. This effect might be explained not only by the improved ability of these liposomes to be taken up by DCs, but also by a more favorable environment for HA to interact with the cell membrane when associated with Chol-containing liposomes. Hemagglutinin in its natural environment (the influenza virus envelope) interacts with Chol rich membranes [35]. For virosomal HA it has been reported that, following endosomal uptake, acidification within the endosome induces HA-mediated fusion (resulting from a conformational change in HA), leading to release of the virosomes into the cytoplasm and a potential MHC class I presentation [36]. Therefore, even though we did not investigate those aspects in our study, it may be that HA itself enhances its own delivery into the cells, leading a potent immune response. 

We focused our investigations on the influence of Chol in the lipid bilayer of different cationic liposomes. Christensen *et al.* [37] compared the immunogenicity of the antigen Ag85B-ESAT-6 combined with cationic liposomes prepared with either the saturated DDA (mixed with the immune modulator D-(+)-trehalose 6,6'-dibehenate [TDB]) or its unsaturated analog dimethyl dioleoyl ammonium bromide (DODA:TDB), which was also suggested as a comparison between rigid and fluid liposomes. The results showed that (gel-state) DDA liposomes were more retained at the injection site than (fluid-state) DODA liposomes, and were better at attracting APCs and inducing a Th1 response. Although we did not investigate the influence of liposome fluidity, the conclusion of Christensen *et al*. differs from what we observed in our work, as we concluded that liposomes based on a liquid organized state (50% Chol) were superior to rigid liposomes. However, it is very difficult to compare these two studies due to some major differences between the components used (e.g., the antigen, the immune modulator) and likely the physico-chemical characteristics (such as size and antigen loading).

In our last experiments, we used Al(OH)_3_ as a reference adjuvant. It is known that aluminum salts promote a Th2 response, and their adjuvant mechanisms is supposedly acting through antigen depot effect, enhancement of antigen uptake by antigen presenting cells (APCs) and the induction of inflammation (known to be activated through local release of uric acid and the triggering of the NALP3 inflammasome) [38,39]. In comparison, the encapsulation of immune modulators in the DC-Chol:DPPC liposomes not only enhanced the overall immune response, but also resulted in a raise of the anti-HA IgG2a/c response. This is in line with a raise of INF-γ secretion by spleen cells (collected from spleens isolated after the *in vivo* study) induced by our formulations (see Supplementary Material, Figure S3). The encapsulation of CpG in nanoparticles has been shown in other studies to enhance the immune response against the co-encapsulated antigen toward a Th1 response [9,13,40,41]. For instance, Joseph *et al*. [42] succeeded to enhance the immunogenicity of a subunit influenza vaccines combined with CpG loaded DMPC:DMPG (dimyristoyl-phosphatidylcholine, dimyristoyl-phosphatidylglycerol) liposomes. Whereas they used 5 µg CpG per dose, we used only 2 µg. Dose reduction might be interesting with regard to the potential side effects of CpG. In contrast with liposomal CpG, free CpG administrated with HA was not effective in our study. This can be explained by the physicochemical properties of CpG for its delivery in soluble form to the intracellularly localized TLR-9 receptor.

Surprisingly, imiquimod, when encapsulated in HA/DC-Chol:DPPC liposomes, did not lead to a better immune response compared to plain HA/DC-Chol:DPPC liposomes. The beneficial activity of TLR-7 ligands was reported in by Geeraedts *et al*. [43], who explained the superior protection induced by H5N1 whole inactivated virus (WIV) compared with subunit or split virus by TLR-7 stimulation from the RNA contained in the WIV vaccine. Therefore, we expected a stronger impact of the TLR-7 agonist imiquimod on the immunogenicity of our influenza subunit vaccine. Weldon *et al*. [44] reported that skin delivery of influenza H1N1 subunit vaccine combined with imiquimod elicited higher levels of serum IgG2a antibody and HI titers as compared to unadjuvanted vaccine in Balb/c mice. They used a similar antigen/imiquimod ratio and imiquimod dose compared to our present study. Besides the different mouse model, the route of administration [45] could be a reason for the discrepancy between their and our results. Moreover, the imiquimod dose used in our study might have been too low. For instance, Rizwan *et al*. [14] succeeded to raise the humoral response against OVA after intramuscular injection in C57Bl/6 mice using liposomes adjuvanted with monophosphoryl lipid A combined with very high doses of imiquimod (150 µg/immunization). Finally, the localization of the imiquimod in the DC-Chol:DPPC liposomal membrane might have limited its interaction with the TLR-7 receptor. Indeed, imiquimod is a hydrophobic molecule [46] and it was mixed with the lipid film before hydration. This might have led to entrapment deep in the lipid bilayer. It would be of interest in future studies to determine to which extent CpG and imiquimod are liberated from the liposomes.

As mentioned earlier, IgG1 and IgG2a/c antibody levels can be used as indicators of a Th2 and Th1 immune response, respectively. As demonstrate in this study, HA needs to be combined with adjuvants to efficiently stimulate both Th1 and Th2 responses, as has also been shown by others [16]. There is growing evidence that both (CD4+) T helper cells and (CD8+) cytotoxic T lymphocytes not only play an important role in controlling viral infection, but also may reduce the severity of disease and decrease mortality [47,48]. The use of cationic liposomes containing immune modulators such as CpG to increase the immunogenicity of the antigen and modulate the immune response towards the Th1 direction is therefore a highly relevant approach. 

## 5. Conclusions

Notwithstanding the clear advantage of using DC-Chol cationic liposomes as an adjuvant for HA, there is still a lack of knowledge regarding their adjuvant mechanism. The data presented here shed some more light on this. First, HA adsorption to the liposome surface was shown to be at least as effective, and an easier way to enhance the immunogenicity of HA, compared to HA encapsulation. Moreover, the presence of Chol in the cationic liposomes appeared to be beneficial for the immune response against HA, an effect that depended on the cationic compound selected. Furthermore, HA was taken up by DCs to a higher extent and was more immunogenic in mice when it was mixed with eDPPC:Chol liposomes compared to eDPPC:DPPC liposomes. Finally, co-delivery of CpG with HA/DC-Chol:DPPC liposomes was found to further promote the immunogenicity of the antigen. In conclusion, cationic liposomes have potential for influenza vaccination provided that the bilayer components and the immune modulator to be encapsulated in these liposomes are carefully selected and their adjuvant mechanism is further investigated.

## Abbreviations

HA: 2 µg HA/dose
Z_ave_: Z-average hydrodynamic diameter
pdi: polydispersity index
ZP: zeta potential
LE HA: loading efficiency hemagglutinin
HA ad: adsorbed HA method
HA enc: encapsulated HA method
LE adj: adjuvant loading efficiency

## Supplementary Files

Supplementary File 1 Supplementary Information (PDF, 1515 KB)
