# Supplementary material for: Adjuvant Effect of Cationic Liposomes for Subunit Influenza Vaccine: Influence of Antigen Loading Method, Cholesterol and Immune Modulators
Source: Pharmaceutics. 2013 Jul 25;5(3):392–410. doi: 10.3390/pharmaceutics5030392 (PMC3836624; doi:10.3390/pharmaceutics5030392)
Supplement: Supplementary File 1 — Supplementary Information (PDF, 1515 KB) [file pharmaceutics-05-00392-s001.pdf]

Supplementary Information

Figure S1. Side scatter-forward scatter (SSC/FSC) dot plots of DCs after 4 h incubation at 4 °C and 37 °C with plain HA and HA/liposome formulations. The gate represents the DC population and excludes cellular debris. The percentages of cells included in the DC gate decreased by *ca*. 10% when the DCs were incubated with cationic liposomes, suggesting a slight toxic effect. No such a decrease was noticed with either HA or (neutral) DPPC:chol liposomes.

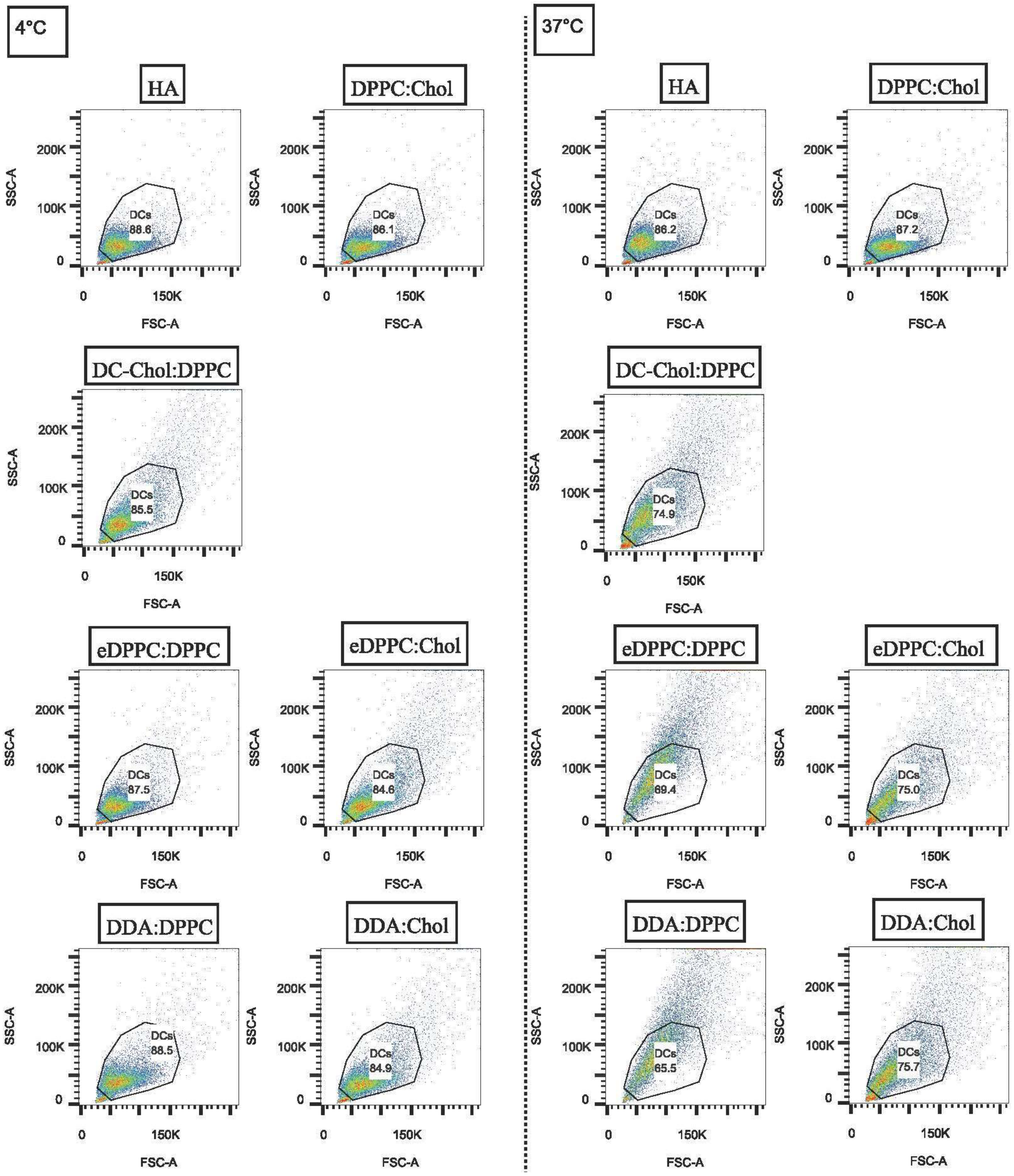

Figure S2. Side scatter-forward scatter (SSC/FSC) dot plots of DCs after 48 h incubation at 37 °C with plain HA, plain HA/DC-Chol:DPPC liposomes and adjuvanted HA/DC-Chol:DPPC liposome formulations. The gate represents the DC population and excludes cellular debris. The percentages of cells included in the DC gate when the DCs were incubated with cationic liposomes were similar to those observed after 4 h (Figure S1).

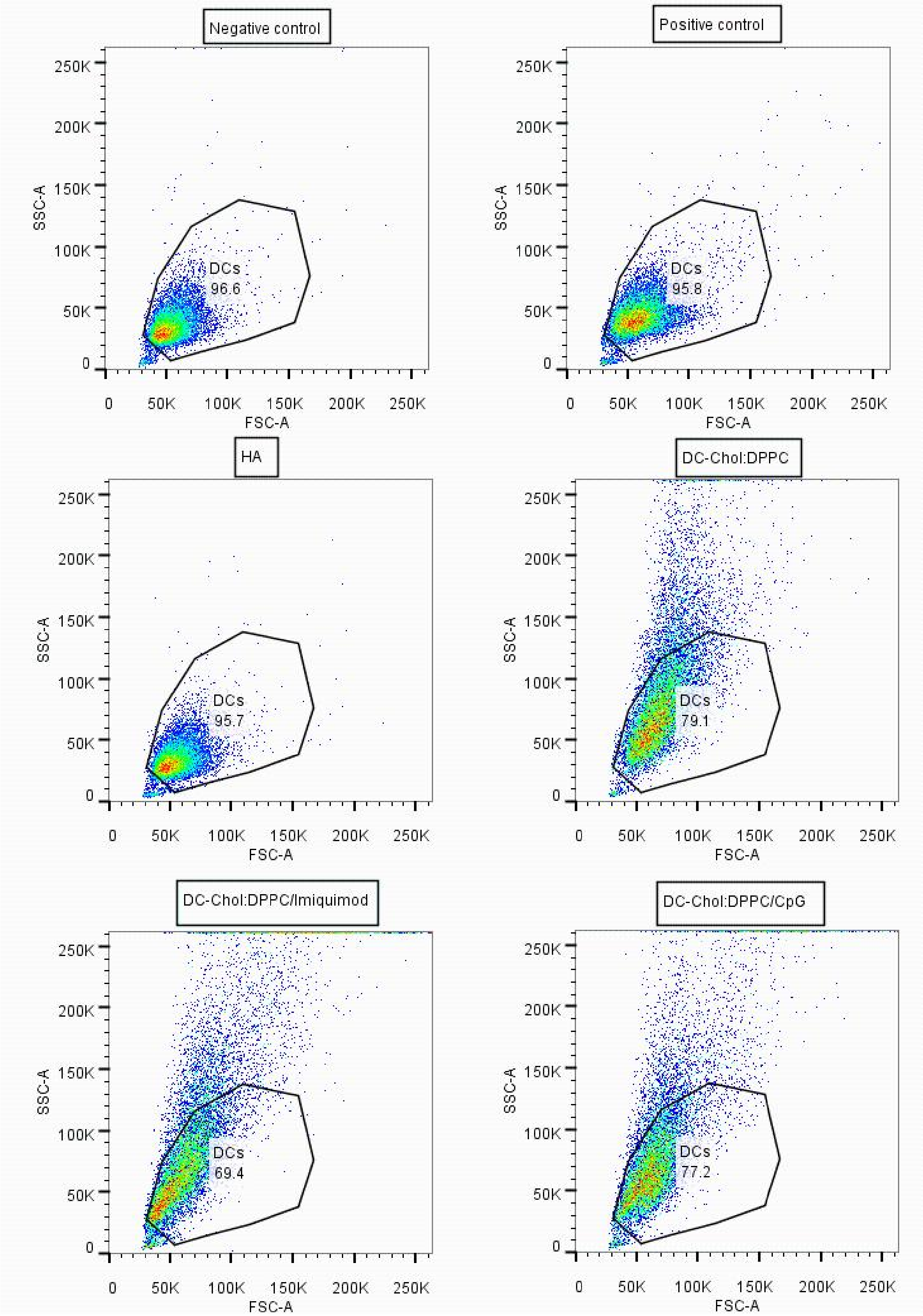

Figure S3. IFN-γ levels secreted by spleen cells collected from mice immunized with 2.0 µg HA, free or mixed with $Al(OH)_3$ or with liposomes containing different immune modulators. The spleens were collected three weeks after the boost immunization, after homogenization the cells were re-stimulated with 5 µg/mL HA, and the release of IFN-γ was determined by ELISA. Significant differences between the liposomal formulations and the $Al(OH)_3$ group are indicated with + ($p < 0.05$).

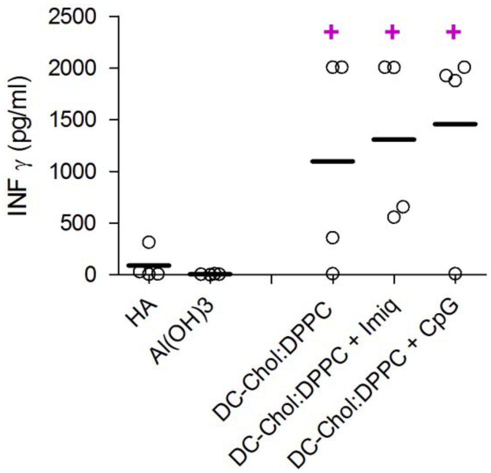

© 2013 by the authors; licensee MDPI, Basel, Switzerland. This article is an open access article distributed under the terms and conditions of the Creative Commons Attribution license (http://creativecommons.org/licenses/by/3.0/).
